# Supplementary material for: Genome-Wide DNA Methylation Profiles in Whole-Blood and Buccal Samples—Cross-Sectional, Longitudinal, and across Platforms
Source: Int J Mol Sci. 2023 Sep 27;24(19):14640. doi: 10.3390/ijms241914640 (PMC10572275; doi:10.3390/ijms241914640)
Supplement: Supplementary file 1 [file ijms-24-14640-s001.zip › ijms-2596237-supplementary figures.pdf]

International Journal of
Molecular Sciences

MDPI

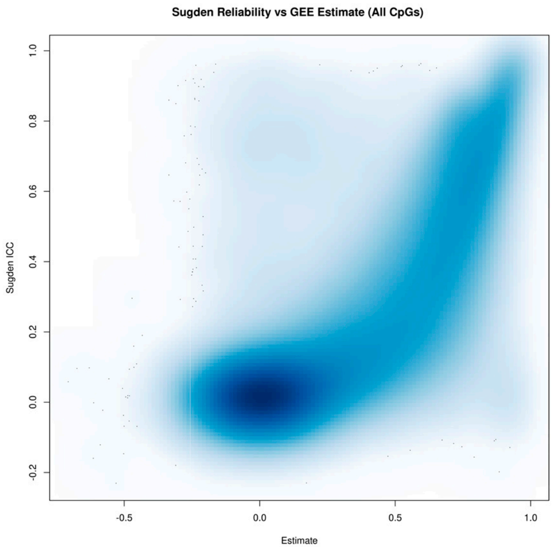

Figure S1. Scatter plot visualizing the relationship between the correlations generated by Sugden et al. and the correlations generated in this study for all CpGs [18]. Across the x-axis are the correlation results obtained in this study by comparing matched samples measured on both the EPIC and 450k array platforms. The y-axis contains the ICC (intra-class correlation) data from Sugden et al., which was also generated from comparing matched whole-blood samples measured on the EPIC and 450k array platforms [18]. The plot includes all CpGs measured in both studies (386,805 CpGs).

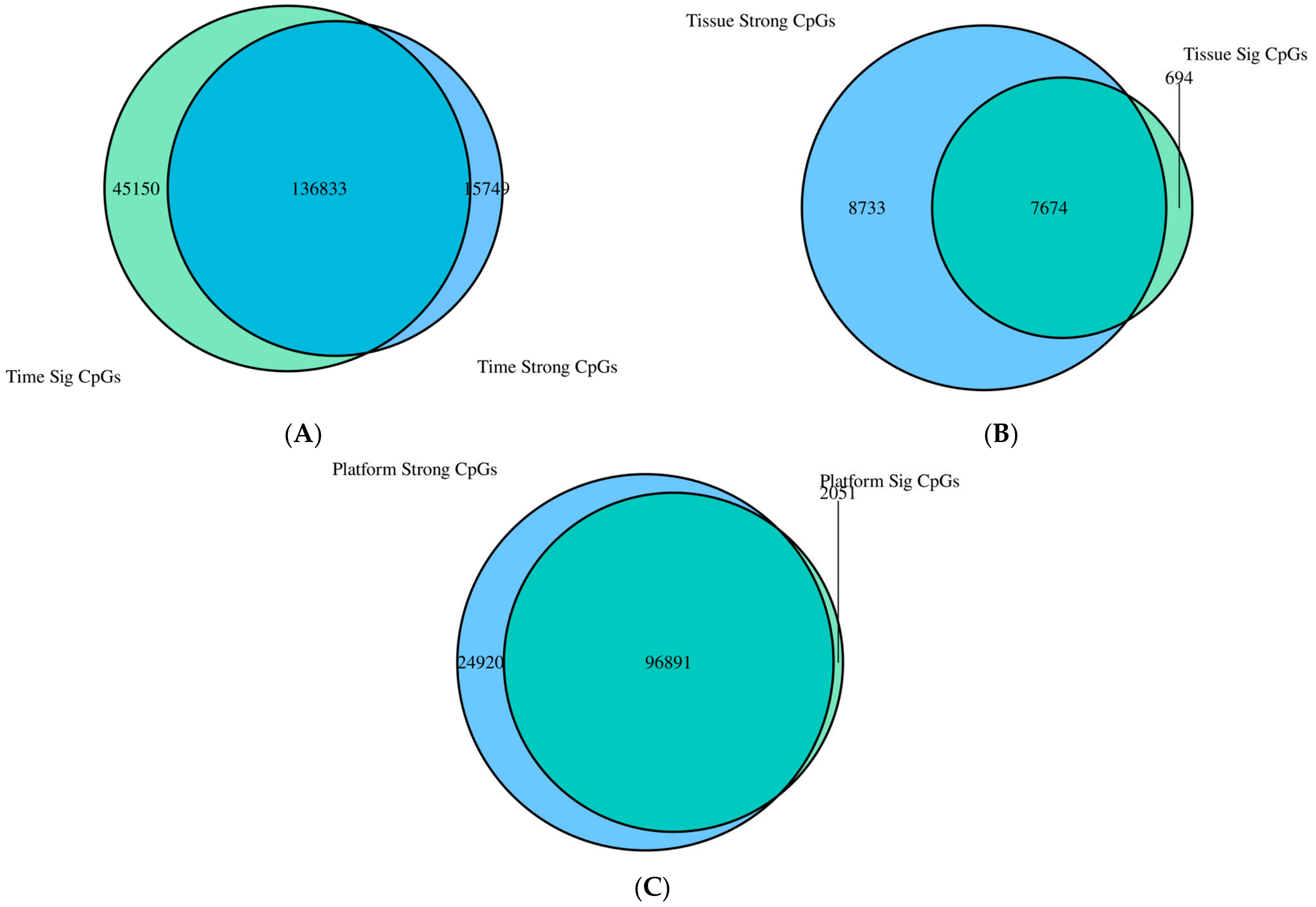

Figure S2. Venn diagrams showing the overlap of significantly and strongly correlated CpGs. In (**A**) longitudinal comparison, (**B**) blood–buccal comparison, (**C**) and EPIC-450K platform comparison.

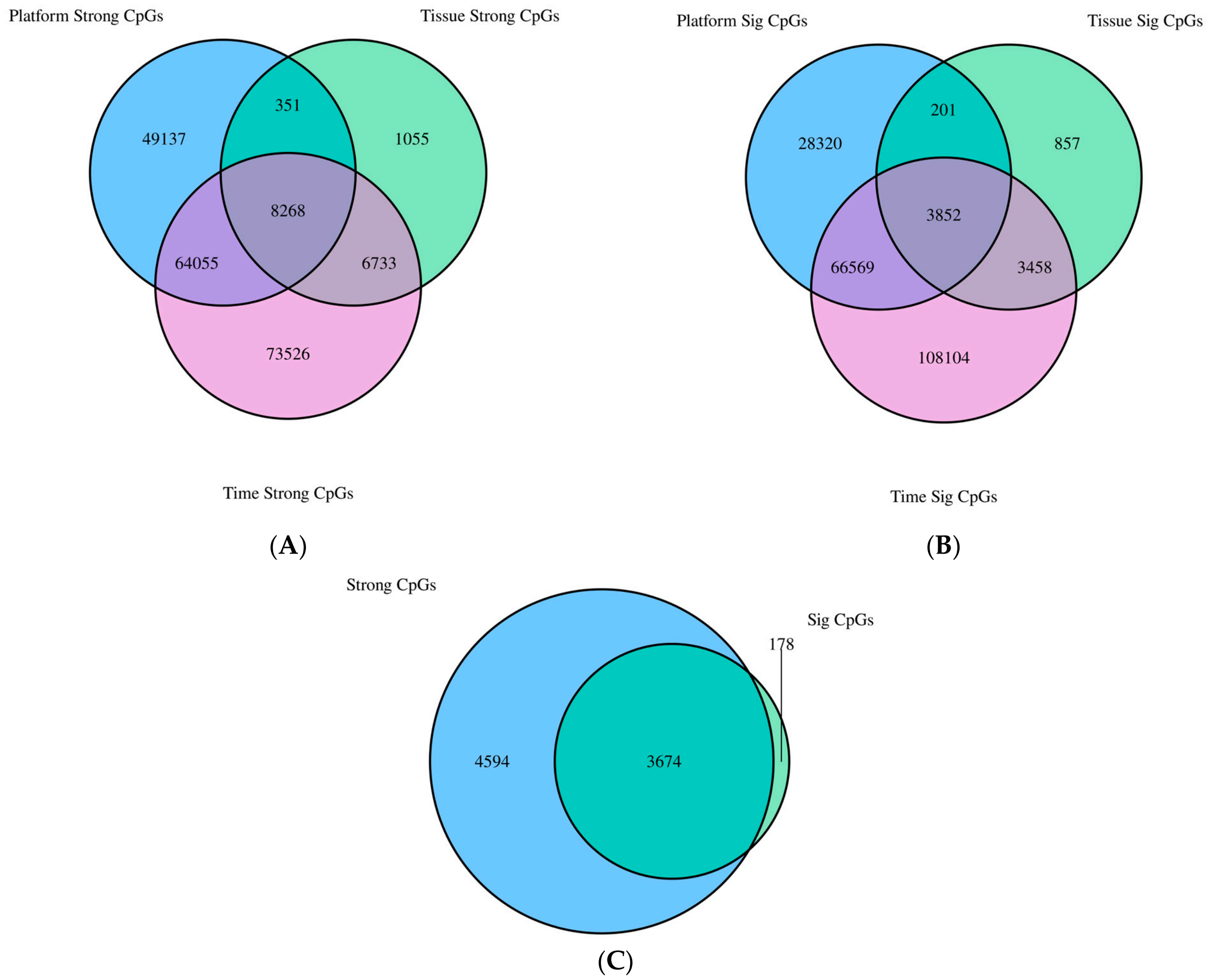

Figure S3. (**A**) Venn diagram showing the overlap of strongly correlated CpGs across all three experiments. (**B**) Venn diagram showing the overlap of significantly correlated CpGs across all three experiments. (**C**) Venn diagram showing the relationship of the strongly correlated and significant CpGs that overlapped across all three experiments.

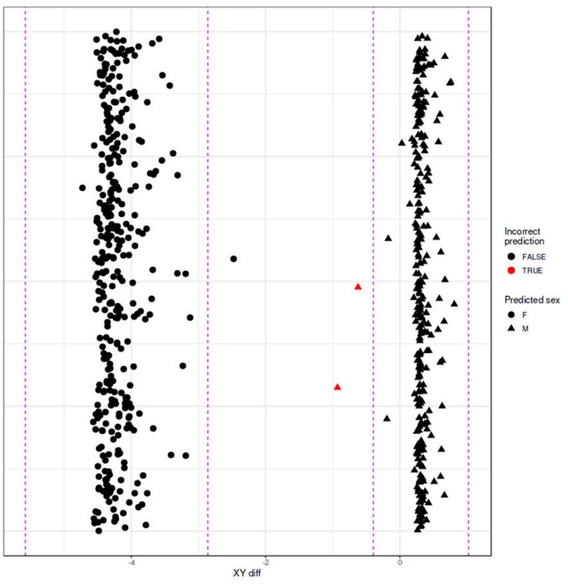

Figure S4. Sex determination of samples via SNP data on an Illumina Infinium EPIC array analyzed via the R package "Meffil". The purple dashed lines represent the thresholds for sex determination set by meffil (standard thresholds were used).

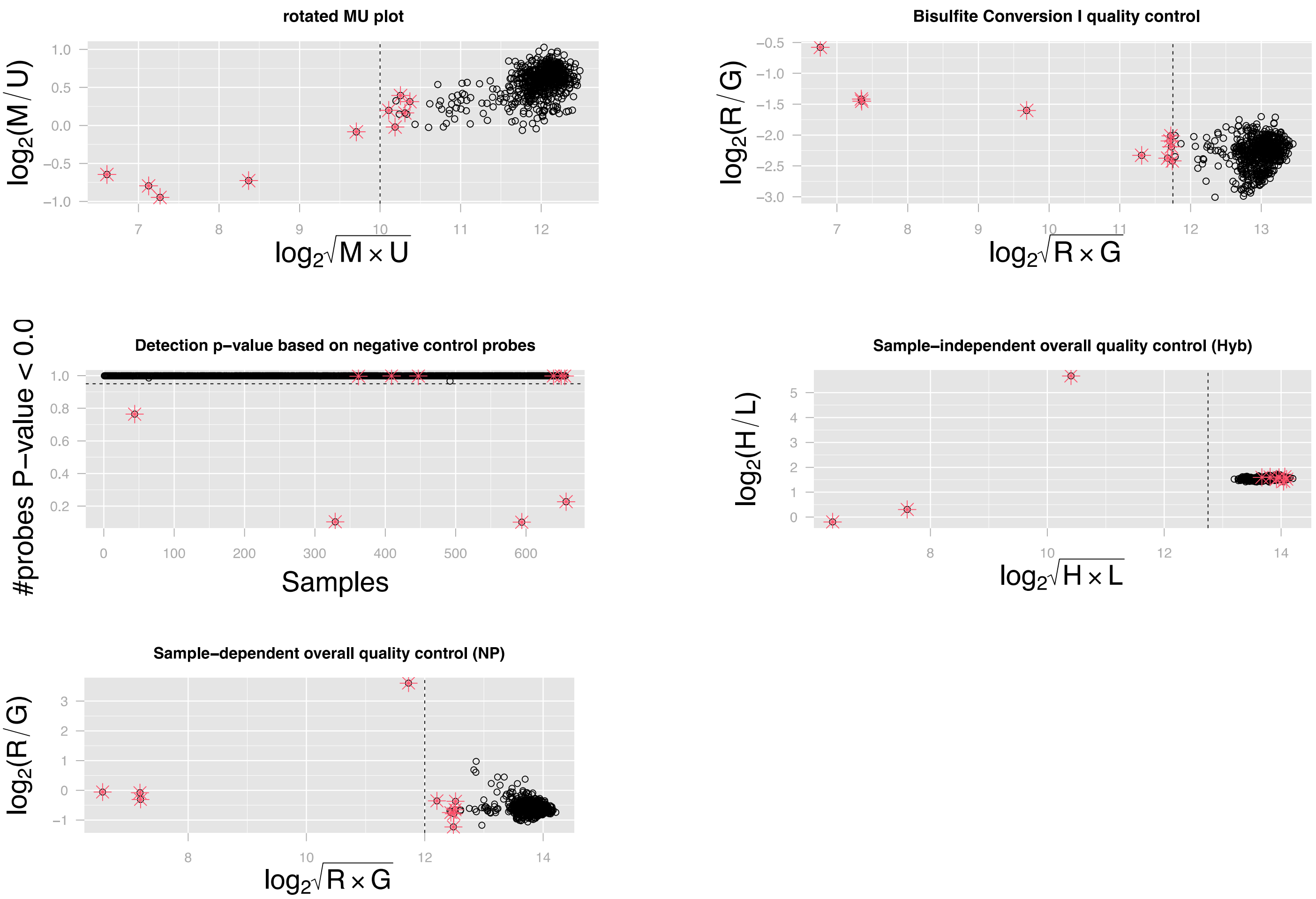

Figure S5. Quality control plots from R package "MethylAid". The dotted lines identify the standard QC thresholds recommended by MethylAid. Samples that did not meet at least one of these thresholds are marked by starring and red shading. The black circles represent samples that passed all QC thresholds.

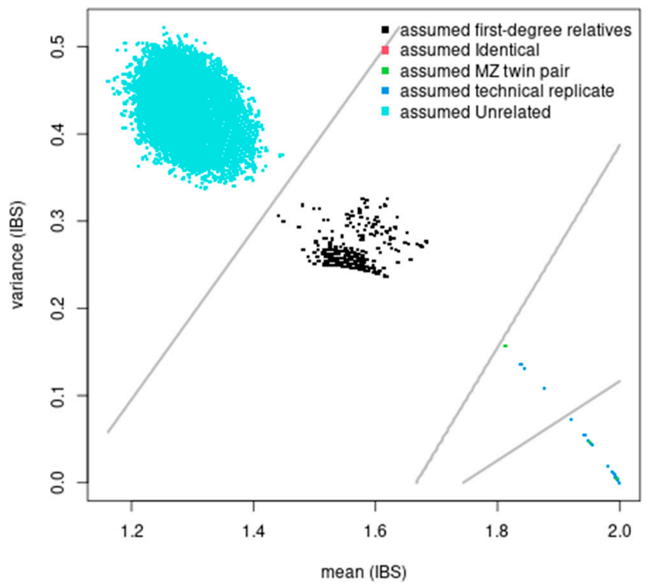

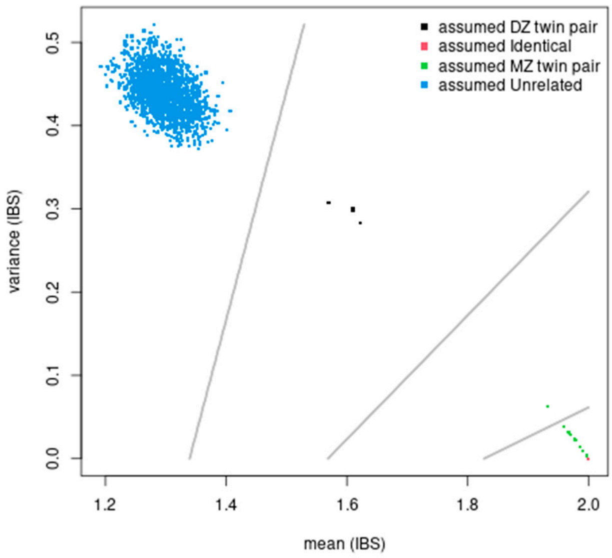

(**A**) (**B**)

Figure S6. IBS determination of sample relations via SNP data on an Illumina Infinium EPIC array analyzed via the R package "omicsPrint". (**A**) Individual relations for all whole-blood samples. (**B**) Individual relations for all buccal samples.

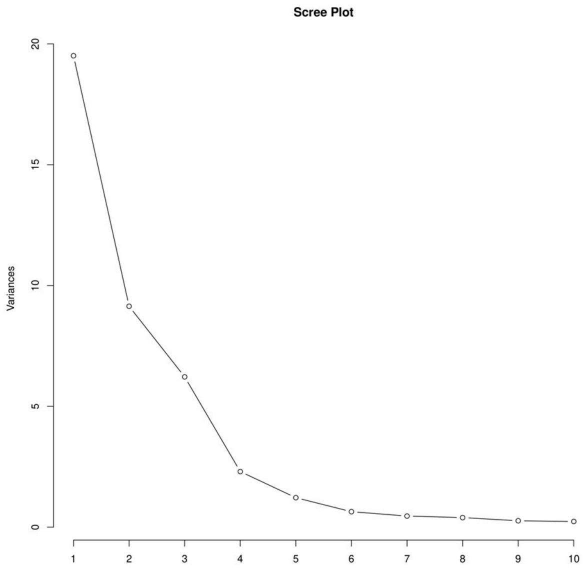

Figure S7. Scree plot visualizing the variance in the control probes explained by each principal component.

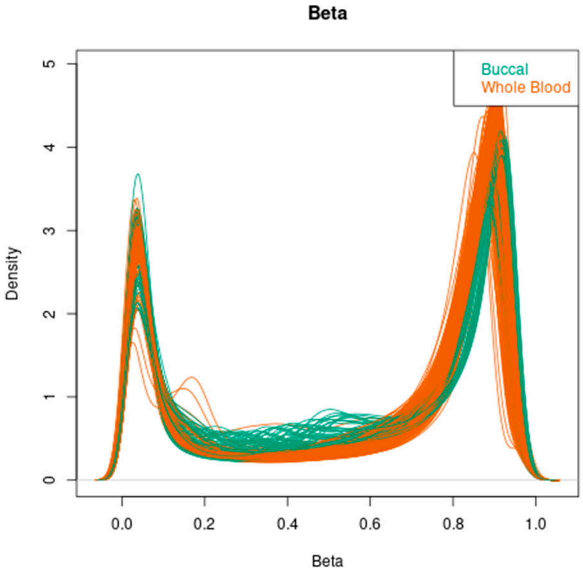

Figure S8. Density plot showing the final distribution of beta values following all data quality control steps. Each line represents an individual sample.

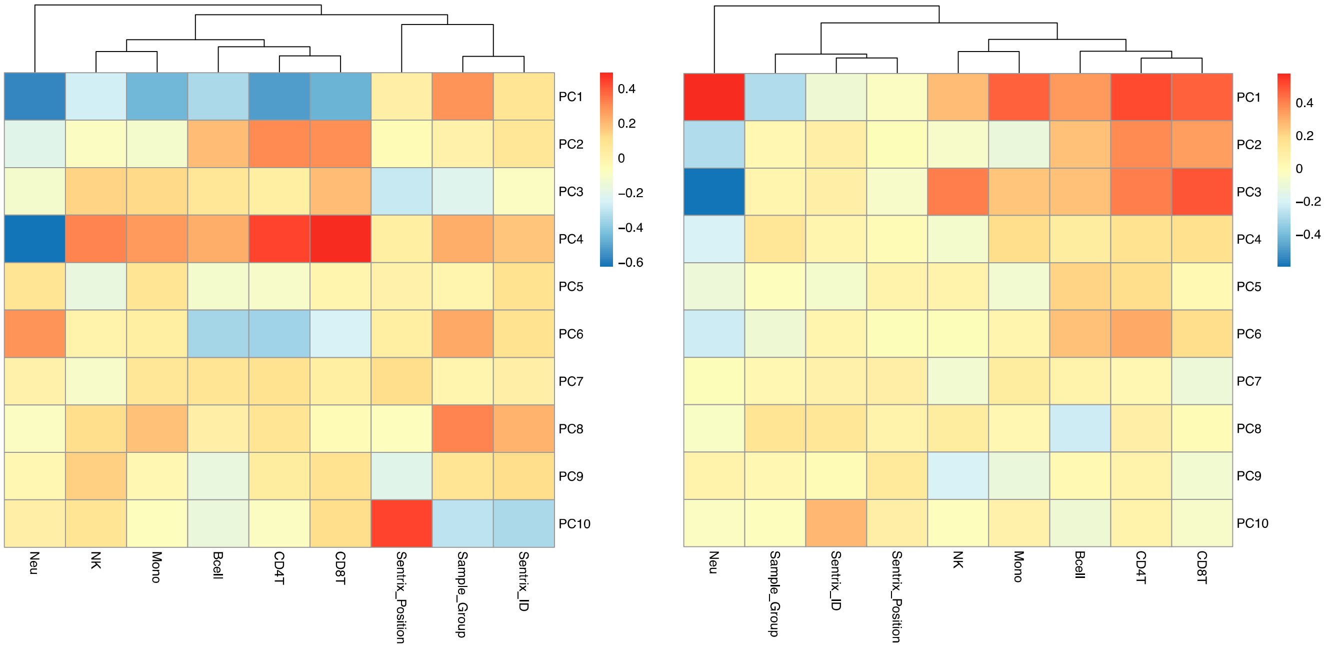

(**A**) (**B**)

Figure S9. Heatmaps showing the associations of the principal components to various technical and biological confounders (**A**) pre-normalization and (**B**) post-normalization.

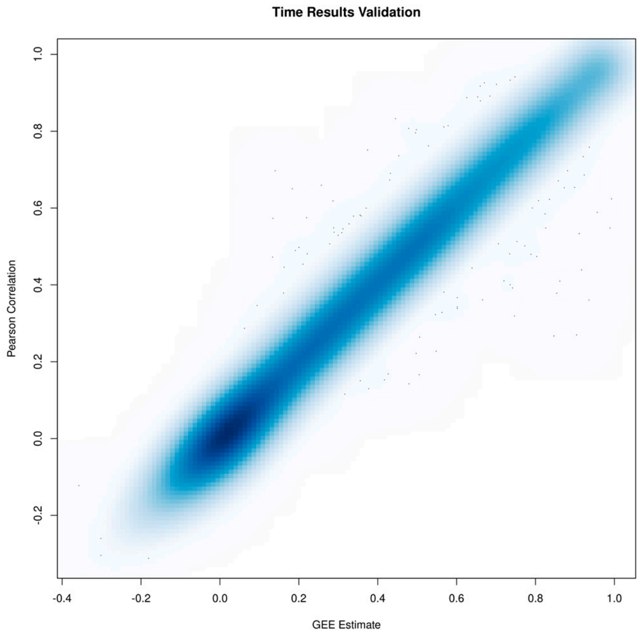

(**A**)

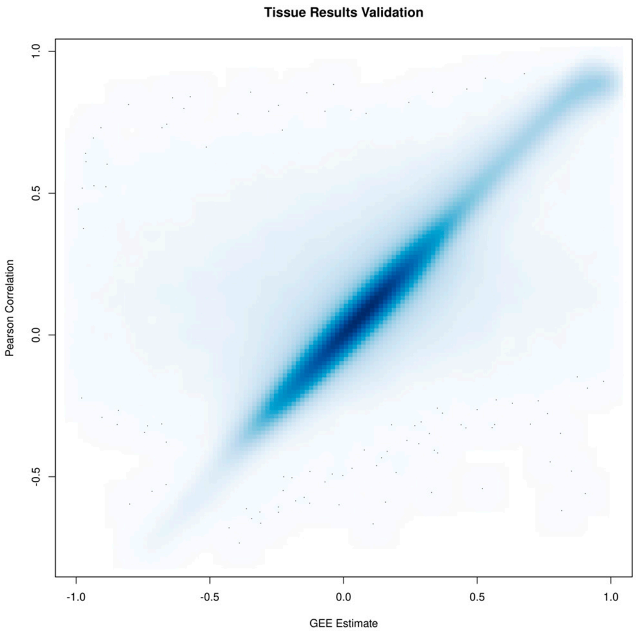

(**B**)

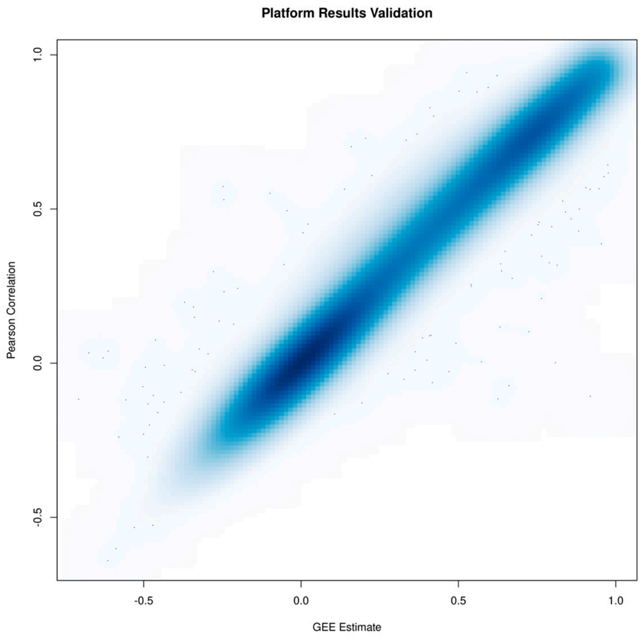

(**C**)

Figure S10. (**A**) Scatter plot comparing the Pearson correlation coefficients to the correlations estimated via GEE for the (**A**) longitudinal comparison, (**B**) comparison between blood and buccal samples, (**C**) and cross-platform comparison (EPIC vs. 450K).
